# Preclinical Evidence and Possible Mechanisms of Baicalein for Rats and Mice With Parkinson's Disease: A Systematic Review and Meta-Analysis

**DOI:** 10.3389/fnagi.2020.00277

**Published:** 2020-09-25

**Authors:** Yu Wang, Na Wei, Xiaoliang Li

**Affiliations:** ^1^Research Institute of Chinese Medicine, Heilongjiang University of Chinese Medicine, Harbin, China; ^2^Key Laboratory of Tropical Translational Medicine of Ministry of Education, Hainan Key Laboratory for Research and Development of Tropical Herbs, School of Pharmacy, Hainan Medical University, Haikou, China

**Keywords:** baicalein, parkinson's disease, preclinical evidence, mechanisms, meta-analysis

## Abstract

Baicalein, a major bioactive flavone of Scutellaria baicalensis Georgi, has neuroprotective properties in several animal models of Parkinson's disease (PD). Here, we conducted a systematic review and meta-analysis to assess the available preclinical evidence and possible mechanisms of baicalein for animal models of PD. Ultimately, 20 studies were identified by searching 7 databases from inception to December 2019. Review Manager 5.3 was applied for data analysis. Meta-analyses showed baicalein can significantly improve neurobehavioral function in animal models with PD, including spontaneous motor activity test (*n* = 5), pole test (*n* = 2), rotarod test (*n* = 9), apomorphine-induced rotations test (*n* = 4), grid test (*n* = 2), and tremor test (*n* = 2). Compared with controls, the results of the meta-analysis showed baicalein exerted a significant effect in increasing the frequency of spontaneous activity, prolongating the total time for climbing down the pole, decreasing the number of rotations, prolongating the descent latency, reducing the amplitude, and the frequency in animal models with PD. The possible mechanisms of baicalein for PD are regulating neurotransmitters, adjusting enzyme activity, antioxidation, anti-inflammatory, inhibiting protein aggregation, restorating mitochondrial dysfunction, inhibiting apoptosis, and autophagy. In conclusion, these findings preliminarily demonstrated that baicalein exerts potential neuroprotective effects through multiple signaling pathways in animal models of PD.

## Introduction

PD is a common and severe degeneration of the central nervous system, which is mainly manifested as bradykinesia, rigidity, and static tremor (Schindlbeck and Eidelberg, 2018), usually accompanying by non-motor symptoms such as depression, sleep disturbances, cognitive decline, etcetera (Schapira et al., 2017). The figures showed that the incidence rate of PD has increased from 3.5 to 42.8% in the past few years (Kovács et al., 2019), affecting about 1% of the population over the age of 60 (Martin et al., 2011). Worse yet, about 1.174 million people died of Parkinson's disease worldwide from 2005 to 2015 (Wang et al., 2016). Although the typical clinical and pathological features of PD have been clear, its etiology and pathogenesis are not fully clarified yet. Over the years, researchers have been exploring the pathogenesis and treatment of PD. Overwhelming evidences demonstrated that levodopa can significantly improve motor function in patients with PD (LeWitt and Fahn, 2016). But it's just about improving the symptoms of PD and cannot stop the progress of the disease (Naskar et al., 2013). Therefore, the search for new effective drugs is a hot topic in PD research. Excitedly, natural medicines characterized with high activity and low side-effect are a valuable resource for us to find compounds against PD.

**Figure 1:**
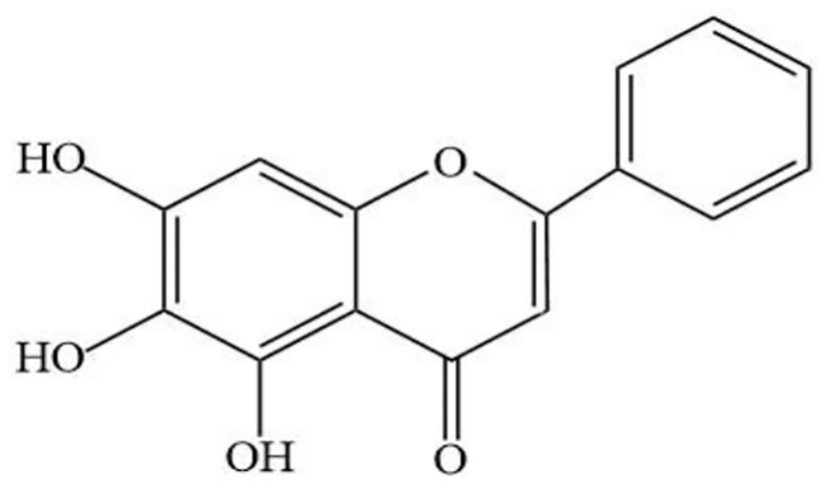
Chemical structure of baicalein.

Baicalein (Figure 1) is a major bioactive flavone, mainly extracted from the root of Scutellaria baicalensis Georgi. Modern pharmacological researches have proved that baicalin has a wide range of biological activities, including antioxidant, anticancer, antiviral, anti-inflammatory, antidiabetic, antithrombotic, hepatoprotective (Sowndhararajan et al., 2017). In addition, recent studies have shown that baicalin has shown strong neuroprotective effects in models of various neurological diseases. *In vivo*, baicalein exerted neuroprotective action through reducing behavioral damage and the depletion of dopaminergic neurons in rotenone-induced PD model (Zhang et al., 2017a). Moreover, other studies have shown that treatment with baicalein significantly attenuated the dopamine (DA) content in striatum induced by 1-methyl-4-phenylpyridinium (MPP^+^) (Hung et al., 2016) and increased the numbers of tyrosine hydroxylase (TH) neurons in PD rat model induced by 6-hydroxydopamine (6-OHDA) (Mu et al., 2009). All these data indicated that baicalin can be used as an effective drug to prevent or treat neurodegenerative diseases such as PD.

The preliminary research basis of animal experiment can provide clearly direction for clinical practice, greatly improving the understanding of clinicians and researchers on the disease mechanism and the progress of intervention measures (Sena et al., 2014). In recent years, many animal studies of baicalein have been reported. However, the efficacy and mechanisms of baicalein for PD have not been systematically appraised and summarized. Thus, in the present study, we performed a systematic review and meta-analysis to assess recent literature on the effects of baicalein therapy on animals with PD and provide current preclinical evidence and potential mechanisms on animal models.

## Materials and Methods

### Data Sources and Search Strategy

The meta-analysis was conducted according to the Preferred Reporting Items for Systematic Review and Meta-Analyses (PRISMA) guidelines (Moher et al., 2015). Seven English and Chinese databases, including China National Knowledge Infrastructure (CNKI), Wanfang Database, Science Direct, PubMed, Web of science, Medline, and EMBASE, were independently searched by two reviewers (XL Li and Y Wang), from their inceptions to December 2019. The following keywords and terms were as follows: “parkinsonian disorders” OR “Parkinson disease” and “Baicalein” OR “Huangqinsu” OR “Baikeli.” All studies were limited to animals.

### Inclusion and Exclusion Criteria

Studies conforming to the following inclusion criteria were selected for this review: (1) participants: experimental animals including mice and rats. (2) invention: baicalein only; (3) outcomes: The effect of baicalein on the animal model of Parkinson's disease, including neurobehavioural. Meanwhile, studies which met the following criteria were excluded: (a) combined use of other drugs; (b) non-animal based studies; (c) case report, comments, clinical experiences, or trials and review article; (d) similar and repeated studies.

### Data Extraction

Two authors (XL Li and Y Wang) extracted data independently from the qualified articles. The following information of each study was recorded: (1) the first author's name, year of publication; (2) animals' characteristics, including species, number, sex, and body weight; (3) methods for PD model establishment and anesthetic used in the model; (4) intervention characteristics, including the dosage and route of administration; (5) main outcome measures and its intergroup differences.

The peak time point was included if the outcomes were tested at different times. The data of the highest dose were extracted when various doses of baicalein were used in study. If the primary data were missing or demonstrated graphically, we tried to contact authors for raw data. And the numerical values in the graph were measured by the digital ruler software when no response was received from the authors.

### Risk of Bias in Individual Studies

The risk of bias in each included study was assessed independently by two investigators (XL Li and Y Wang) using the Systematic Review Centre for Laboratory animal Experimentation (SYRCLE)'s risk of bias tool for animal studies, which was used to evaluate bias in six domains: selection bias (sequence generation, baseline characteristics, and allocation concealment), performance bias (random housing and blinding), detection bias (random outcome assessment and blinding), attrition bias, reporting bias, and other biases (Hooijmans et al., 2014).

### Statistical Analysis

RevMan V.5.3 software was applied for Meta-analyses. Outcome measures were all treated as continuous data and expressed as standardized mean difference (SMD) with 95% confidence interval (CI). Heterogeneity among individual studies was assessed using the I-square (*I*^2^) statistics test. If *I*^2^ > 50%, a random effect model was adopted. Instead, a fixed effect model was used. Publication bias was assessed by funnel plots and Egger's test. Probability value *P* < 0.05 was considered statistically significant.

## Results

### Study Selection

**Figure 2:**
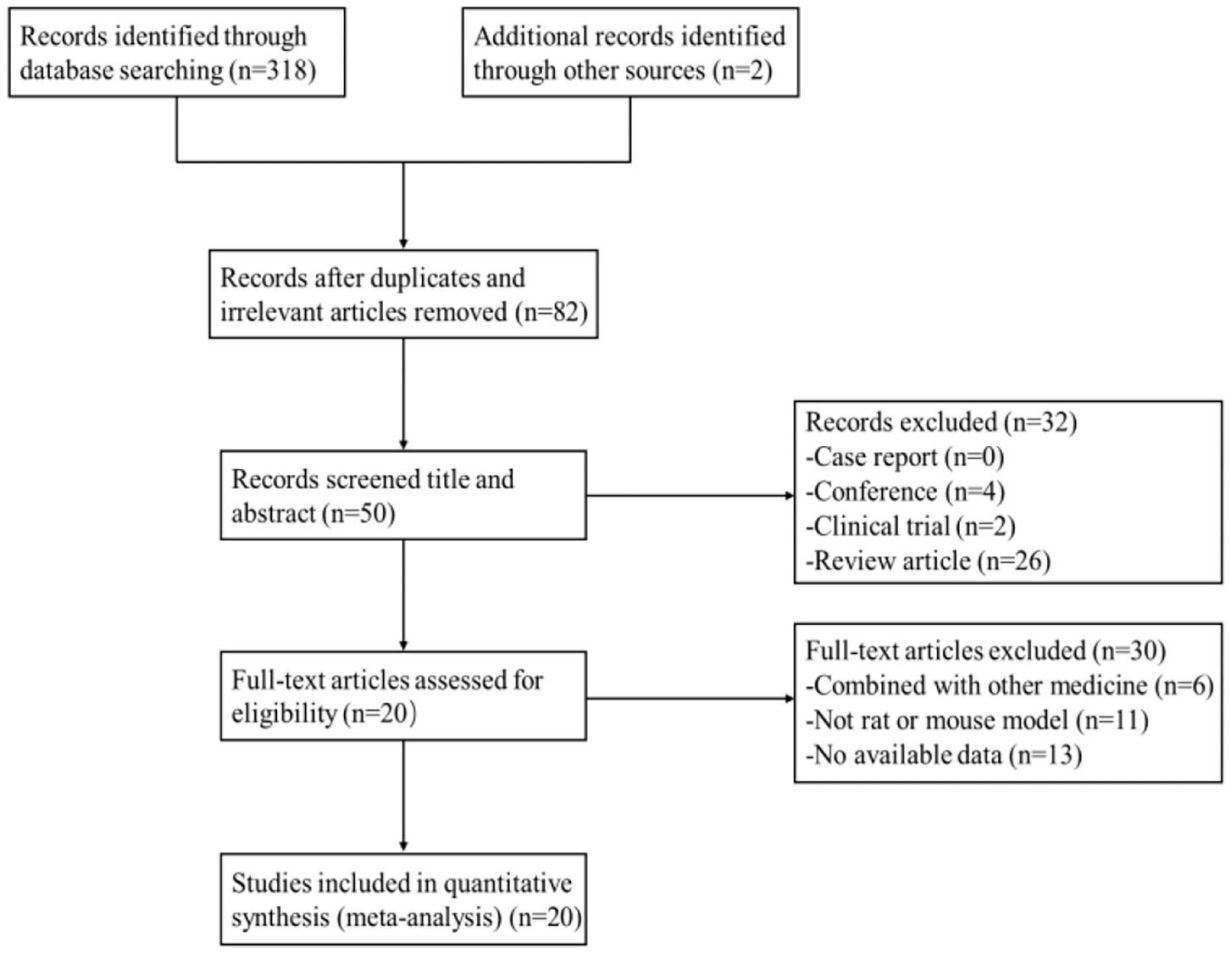
Summary of the process for identifying candidate studies.

After primary search from seven databases, a total of 320 potentially publications were identified. After removal of repetitive and irrelevant articles, 82 records were remained. By screening titles and abstracts, 32 studies were excluded because they were conferences, clinical trials, or review articles. Then secondary screening was performed by reading the remaining full-text articles, and 30 studies were excluded for at least one of the following reasons:(1) no available data; (2) not rat or mouse model; (3) combined with other medicine. Eventually, 20 eligible studies were selected. A flow diagram of the study selection process is shown in Figure 2.

### Characteristics of Included Studies

Twenty rats or mice experiments between 2008 and 2019 were included. Sixteen studies were published in English, and four studies were published in Chinese which containing one online PhD theses. For animal species, Sprague-Dawley (SD) rats were used in 9 studies, C57B/6 J mice were used in 8 studies, ICR mice were used in 2 studies, and the remaining 1 study used Kunming (KM) mice. All animals were male in the included studies. The body weight of SD rats ranged from 180 to 350 g, while the body weight of mice ranged from 18 to 30 g. Anesthetic was reported in 11 studies, including sodium pentobarbital (*n* = 4), chloralose (*n* = 1), equithesin (*n* = 1), ethyl ether (*n* = 1), halothan (*n* = 1), and chloral hydrate (*n* = 4). PD models were established by using 1-methyl-4-phenyl-1,2,3,6-tetrahydropyridine (MPTP) (*n* = 6), 6-OHDA (*n* = 6), rotenone (*n* = 5), MPP^+^ (*n* = 1), acrolein (*n* = 1), oxotremorine (*n* = 1). There were ways of administration of baicalein, including intragastric administration (*n* = 9), intraperitoneal administration (*n* = 5), subcutaneous administration (*n* = 1). The changes of praxeology as primary outcome for assessing baicalein were carried out by the spontaneous motor activity test (*n* = 5), pole test (*n* = 2), apomorphine-induced rotation test (*n* = 4), rotarod test (*n* = 9), grid test (*n* = 2), and tremor test (*n* = 6). About the changes of neurotransmitters, DA was reported in 12 studies, 5-Hydroxyindole-3-acetic acid (5-HIAA) in 2 studies, 5-HT in 2 studies, (3,4-dihydroxyphenylacetic acid) DOPAC in 8 studies, E in 2 studies, gamma-aminobutyric acid (GABA) in 2 studies and glutamate (GLU) in 2 studies. In addition, TH was reported in 13 studies, Cathepsin B in 2 studies, ED-1 in 2 studies, glial fibrillary acidic protein (GFAP) in 4 studies, a-Caspase 1 in 2 studies, superoxide dismutase (SOD) in 2 studies, malondiadehycle (MDA) 5 in studies, glutathione peroxidase (GSH-Px) in 3 studies. The detailed characteristics of the included studies are summarized in Table 1.

**Table 1:** Characteristics of the 20 included animal studies.

**Study (years)**	**Species (Sex, experimental/control group)**	**Weight**	**Modeling approach**	**Anesthetic**	**Treatment group**	**Control group**	**Outcome measure**	**Intergroup differences**
Zhang et al., 2017b	Male sprague-dawley rats(10/15)	240–260 g	Rotenone	–	Baicalein (100, 200, 400 mg/kg p.o.)	–	Spontaneous motor activity test Rotarod test TH	*P* < 0.05 *P* < 0.05 *P* < 0.01
Hung et al., 2016	Male sprague-dawley rats (-/-)	300–350 g	MPP+	Chloral hydrate	Baicalein (10 and 30 mg/kg)	10% DMSO in saline	DA TH IL-1β ED-1 a-Caspase 1 Cathepsin B	*P* < 0.05 *P* < 0.05 *P* < 0.05 *P* < 0.05 *P* < 0.05 *P* < 0.05
Mu et al., 2009	Male sprague-dawley rats(12/12)	180–200 g;	6-OHDA	3% sodium pentobarbital (45 mg/kg i.p.)	Baicalein (200 mg/kg; i.g.)	Saline i.g.	Apomorphine-induced rotation test Tremor test -Burst frequency -Burst amplitude TH GFAP	P > 0.05 *P* < 0.01 *P* < 0.01 *P* < 0.05 *P* < 0.01
Cheng et al., 2008	Male C57BL/6 mice (10/10)	~25 g	MPTP	–	Baicalein (200 mg/kg; i.g.)	Saline i.g.	Spontaneous motor activity test Pole test DA DOPAC 5-HT 5-HIAA TH GFAP SOD GSH-Px MDA	*P* < 0.05 *P* < 0.05 *P* < 0.01 P > 0.05 *P* < 0.05 P > 0.05 *P* < 0.05 *P* < 0.01 *P* < 0.05 *P* < 0.05 *P* < 0.05
He et al., 2015	Male sprague-dawley rats(12/12)	180–200 g	6-OHDA	3% sodium pentobarbital (50 mg/kg i.p.)	Baicalein (200 mg/kg i.g.)	Distilled water i.g.	Spontaneous motor activity test Apomorphine-induced rotation test Tremor test -Burst frequency -Burst amplitude DA DOPAC TH	*P* < 0.05 *P* < 0.01 *P* < 0.05 *P* < 0.01 *P* < 0.05 P > 0.05 *P* < 0.05
Li, 2011	Male sprague-dawley rats ^(−/−)^	180–200 g	6-OHDA	3% sodium pentobarbital (50 mg/kg i.p.)	Baicalein (200 mg/kg; i.g.)	–	Spontaneous motor activity test Rotarod test Apomorphine-induced rotation test Tremor test-burst frequency DA DOPAC 5-TH 5-HIAA GABA GLU E TH GFAP	*P* < 0.05 *P* < 0.05 *P* < 0.01 *P* < 0.001 P > 0.05 *P* < 0.05 P > 0.05 P > 0.05 P > 0.05 P > 0.05 P > 0.05 *P* < 0.05 *P* < 0.05
Mu et al., 2011	Male C57BL/6 mice (12/12)	~25 g	MPTP	–	Baicalein (140, 280, 560 mg/kg i.g.)	Saline i.g.	Spontaneous motor activity test Rotarod test DA DOPAC TH SOD GSH-P x MDA	*P* < 0.05 *P* < 0.05 *P* < 0.05 P > 0.05 *P* < 0.05 *P* < 0.05 *P* < 0.05 *P* < 0.05
Gao et al., 2015	Male C57BL/6 mice (15/15)	–	MPTP	–	Baicalein (140 and 280 mg/kg i.g.);	Saline i.g.	Pole test Rotarod test	*P* < 0.001 *P* < 0.01
Im et al., 2005	Male ICR mice ^(−/−)^	26–28 g	6-OHDA	–	Baicalein (25 and 50 mg/kg, i.p)	5% DMSO	Rotarod test DA DOPAC TH MDA	*P* < 0.05 *P* < 0.05 *P* < 0.05 *P* < 0.05 *P* < 0.05
Xue et al., 2014	Male C57BL/6 mice ^(−/−)^	18–24 g	MPTP	halothan	Baicalein (10 mg/kg, i.p.)	Saline i.p.	Rotarod test TNF-a IL-1β	*P* < 0.05 *P* < 0.05 *P* < 0.05
Lee et al., 2014	Male C57BL/6 mice ^(−/−)^	20–23 g	MPTP	Ethyl ether	Baicalein (1 and 10 mg/kg i.p.);	PBS containing 5% ethanol and 2% Tween 20 i.p.	Rotarod test TH	*P* < 0.01 *P* < 0.01
Hu et al., 2016	Male C57BL/6 mice ^(−/−)^	–	Rotenone	–	Baicalein (100 mg/kg; i.p.)	–	Rotarod test Grid test DA DOPAC TH	*P* < 0.01 *P* < 0.01 *P* < 0.01 *P* < 0.01 *P* < 0.05
Kuang et al., 2017	Male C57BL/6 mice ^(−/−)^	–	Rotenone	–	Baicalein (100 mg/kg; i.p.)	–	Rotarod test Grid test DA	*P* < 0.01 *P* < 0.01 *P* < 0.01
Im et al., 2006	Male ICR mice ^(−/−)^	26–28g	6-OHDA	Equithesin (0.6 mg/ml, 5 ml/kg, i.p.)	Baicalein (25 and 50 mg/kg, i.p)	5% DMSO	Apomorphine-induced rotation test DA DOPAC MDA TH	*P* < 0.05 *P* < 0.05 *P* < 0.05 *P* < 0.05 *P* < 0.05
Yu et al., 2012	Male sprague-dawley rats(12/12)	180–200 g	6-OHDA	Sodium pentobarbital (45 mg/kg i.p.); Chloralose (300 mg/kg, i.p.)	Baicalein (100, 200 and 400 mg/kg i.g.);	Saline i.g.	Tremor test -Burst frequency -Burst amplitude DA GABA GLU	*P* < 0.01 *P* < 0.001 P > 0.05 *P* < 0.05 *P* < 0.05
Yang et al., 2018	Male KM mice (8/8)	18–22 g	Oxotremorine	–	Baicalein (120, 240, 480 mg/kg i.g.)	Saline i.g.	DA DOPAC E GSH-P x MDA	*P* < 0.01 *P* < 0.01 P > 0.05 P > 0.05 P > 0.05
Zhao et al., 2018	Male sprague-dawley rats ^(−/−)^	300–350 g	Acrolein	Chloral hydrate (450 mg/kg)	Baicalein (30 mg/kg)	10% DMSO in saline	ED-1 a-Caspase 1 Cathepsin B DA IL-1β GFAP	*P* < 0.05 *P* < 0.05 *P* < 0.05 *P* < 0.05 *P* < 0.05 *P* < 0.05
Han et al., 2019	Male Sprague–Dawley rats(12/12)	200 ± 20 g	Rotenone	Chloral hydrate (300 mg/kg)	Baicalein (200 mg/kg s.c.)	Saline s.c.	TH	*P* < 0.01
Zheng et al., 2019	Male C57BL/6 mice (6/6)	25–30 g	MPTP	–	Baicalein (10 mg/kg i.p.)	Saline	TH	P > 0.05
Zhang et al., 2017a	Male Sprague-Dawley rats(10/10)	240–260 g	Rotenone	–	Baicalein (100, 200, 400 mg/kg p.o.)	–	GFAP TNF-a IL-1β	*P* < 0.01 *P* < 0.05 P > 0.05

### Study Quality

**Figure 3:**
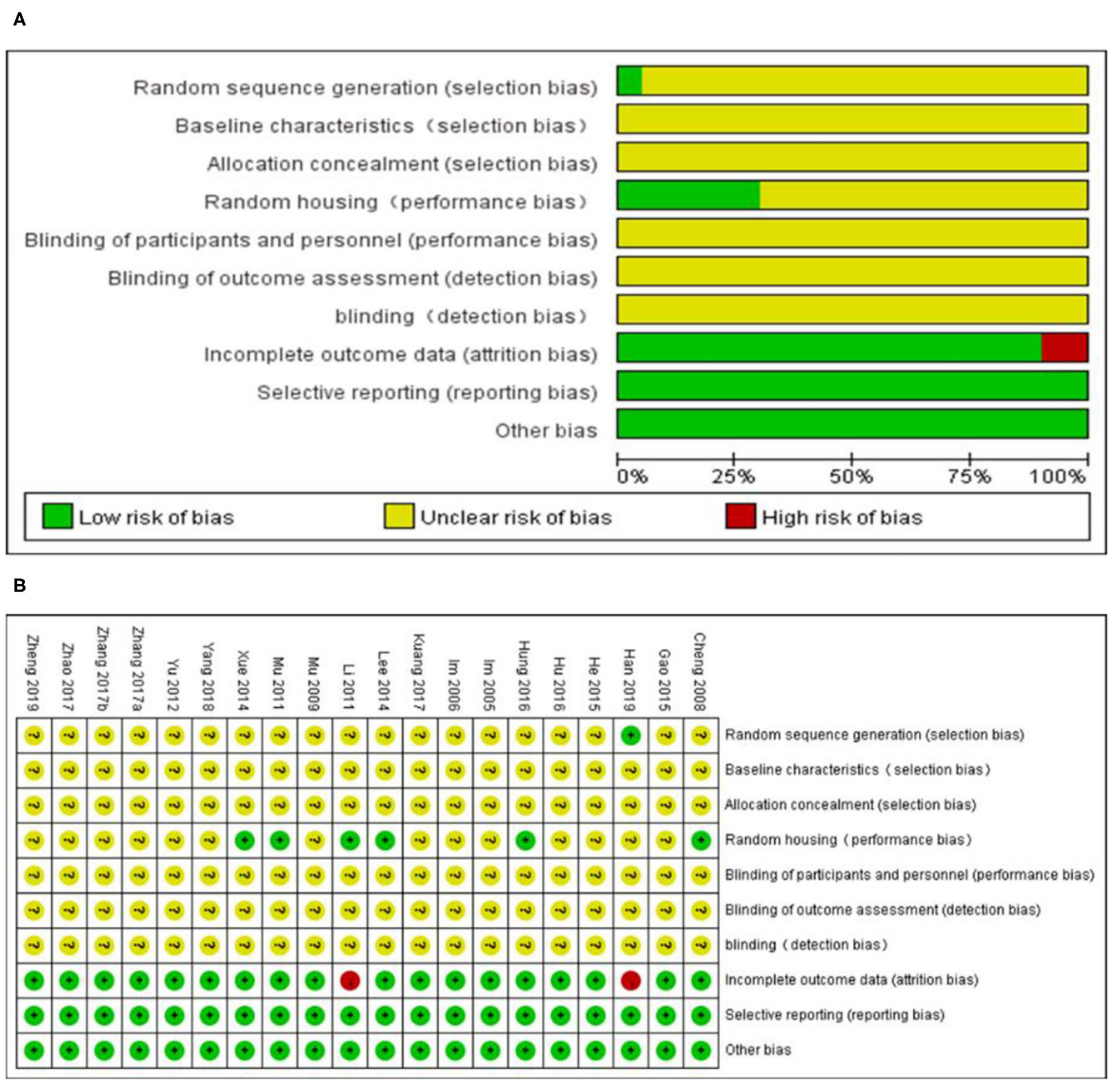
Risk of bias of the included studies. **(A)** Risk of bias graph. **(B)** Risk of bias summary. + = low risk of bias, – = high risk of bias, ? = unclear risk of bias.

The overall quality of the included studies was relatively low (Figure 3). All but one study was not reported randomization. All studies included were unclear in baseline characteristics, allocation concealment, blinding of participants, and detection bias. while, these studies were all provided information regarding selective reporting and other bias. Six studies adequately reported random housing and 16 studies adequately reported incomplete outcome data.

### Effectiveness

#### Behavior Function

**Figure 4:**
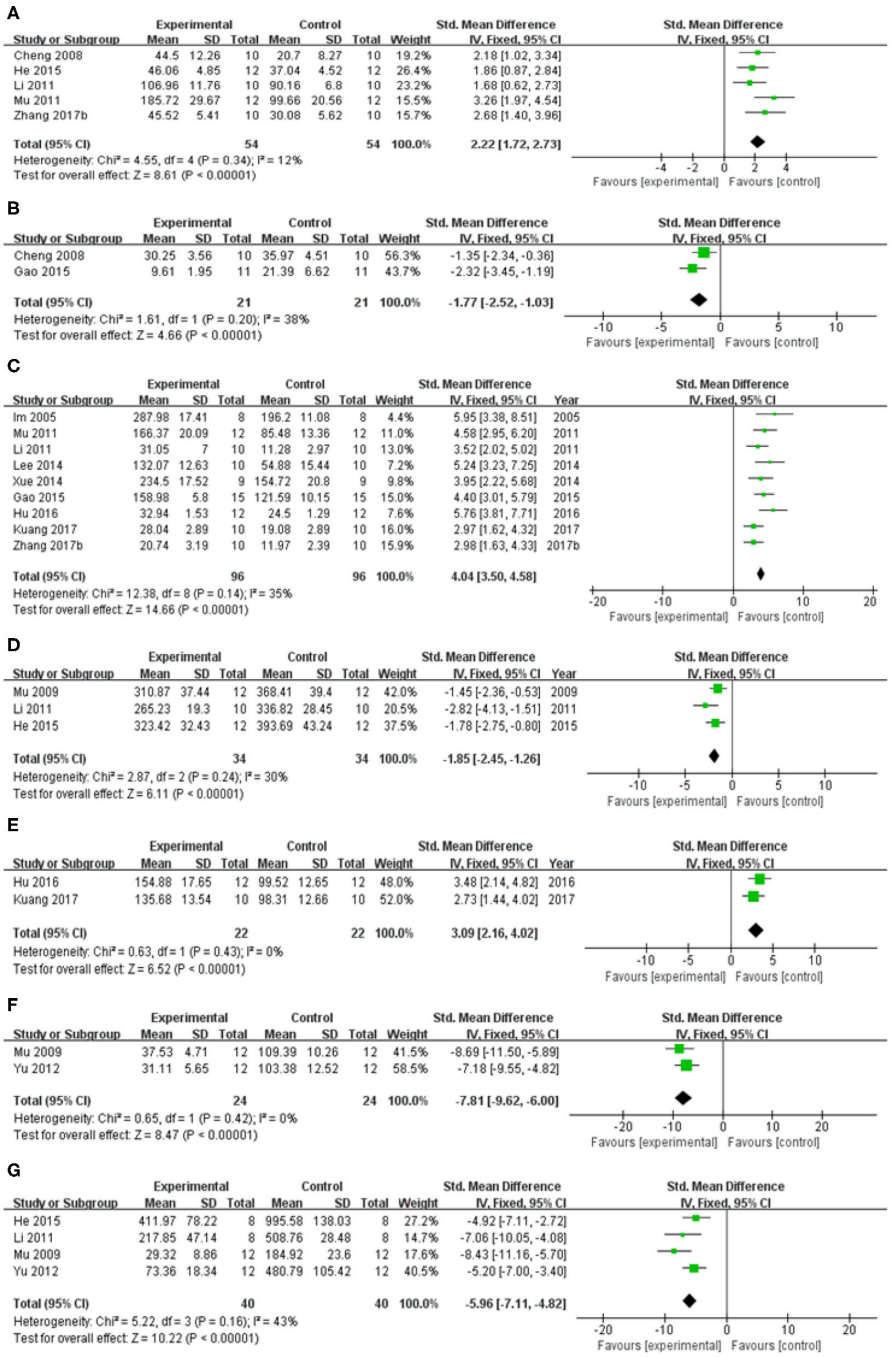
Forest plot of studies investigating the effect of baicalein on animal behavior. **(A)** Spontaneous motor activity test, **(B)** Pole test, **(C)** Rotarod test, **(D)** Apomorphine-induced rotations test, **(E)** Grid test, **(F)** altitude, and **(G)** frequency in tremor test compared with control group.

In spontaneous motor activity test, meta-analysis of 5 studies (Cheng et al., 2008; Li, 2011; Mu et al., 2011; He et al., 2015; Zhang et al., 2017b) showed significant effect of baicalein for increasing the frequency of spontaneous activity in PD animals compared with control group [*n* = 54, SMD = 2.22, 95% CI (1.72–2.73), *P* < 0.00001; heterogeneity: χ = 4.55, df = 4 (*P* = 0.34); *I*^2^ = 12%; Figure 4A]. In pole test, meta-analysis of 2 studies (Cheng et al., 2008; Gao et al., 2015) showed significant effect of baicalein for prolongating the total time for climbing down the pole in PD animals compared with control group [*n* = 21, SMD = −1.77, 95% CI (−2.52 to −1.03), *P* < 0.00001; heterogeneity: χ = 1.61, df = 1 (*P* = 0.20); *I*^2^ = 38%; Figure 4B]. In rotarod test, meta-analysis of 9 studies (Im et al., 2005; Li, 2011; Mu et al., 2011; Lee et al., 2014; Xue et al., 2014; Gao et al., 2015; Hu et al., 2016; Kuang et al., 2017; Zhang et al., 2017b) showed significant effect of baicalein for extending the time spent on the rod in PD animals compared with control group [*n* = 96, SMD = 4.04, 95% CI (3.50–4.58), *P* < 0.00001; heterogeneity: χ = 12.38, df = 8 (*P* = 0.14); *I*^2^ = 35%; Figure 4C]. In apomorphine-induced rotations test, meta-analysis of 4 studies (Im et al., 2006; Mu et al., 2009; Li, 2011; He et al., 2015) showed significant effect of baicalein for decreasing in the number of apomorphine-induced rotations in PD animals compared with control group [*n* = 42, SMD = −1.96, 95% CI (−2.55 to −1.37), *P* < 0.00001; heterogeneity: χ = 21.68, df = 3 (*P* < 0.00001); *I*^2^ = 86%]. Through sensitivity analysis, we removed one study (Im et al., 2006) which utilized ICR mice with PD, while the other studies used models established with SD rats. Meta-analysis of 3 studies (Mu et al., 2009; Li, 2011; He et al., 2015) showed significant effect of baicalein for decreasing in the number of apomorphine-induced rotations in PD animals compared with control group [*n* = 34, SMD = −1.85, 95% CI (−2.45 to −1.26), *P* < 0.00001; heterogeneity: χ = 2.87, df = 2 (*P* = 0.24); *I*^2^ = 30%; Figure 4D]. In grid test, meta-analysis of 2 studies (Hu et al., 2016; Kuang et al., 2017) showed significant effect of baicalein for prolongating the descent latency in PD animals compared with control group [*n* = 22, SMD = 3.09, 95% CI (2.16–4.02), *P* < 0.00001; heterogeneity: χ = 0.63, df = 1 (*P* = 0.43); *I*^2^ = 0%; Figure 4E]. In tremor test, meta-analysis of 2 studies (Mu et al., 2009; Yu et al., 2012) showed significant effect of baicalein for reducing the amplitude in PD animals compared with csontrol group [*n* = 24, SMD = −7.81, 95% CI (−9.62 to −6.00), *P* < 0.00001; heterogeneity: χ = 0.65, df = 1 (*P* = 0.42); *I*^2^ = 0%; Figure 4F]. Meta-analysis of 4 studies (Mu et al., 2009; Li, 2011; Yu et al., 2012; He et al., 2015) showed significant effect of baicalein for reducing the frequency in PD animals compared with control group [*n* = 40, SMD = −5.96, 95% CI (−7.11 to −4.82), *P* < 0.00001; heterogeneity: χ = 5.22, df = 3 (*P* = 0.16); *I*^2^ = 43%; Figure 4G]. These results suggested that baicalein ameliorated behavioral deficits in PD animals.

#### Neuroprotective Mechanism

##### The adjustment of neurotransmitters disequilibrium

**Figure 5:**
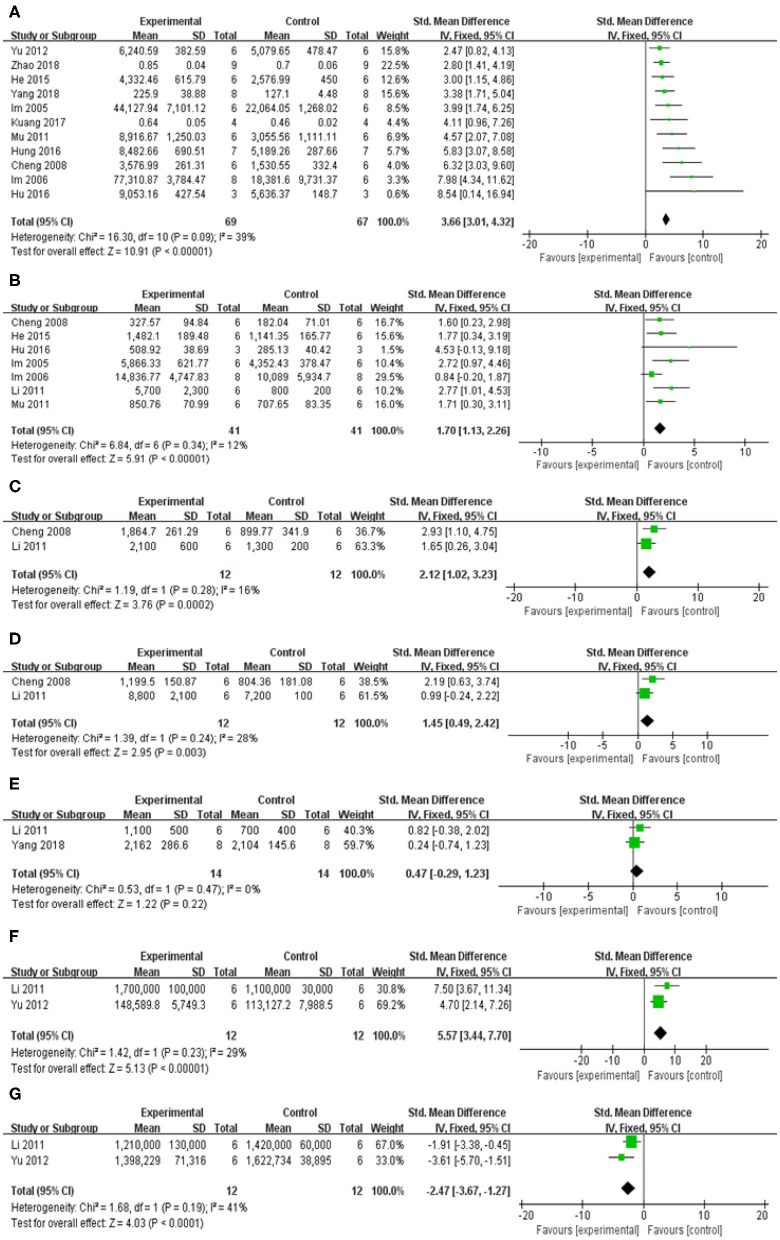
Forest plot of studies investigating the effect of baicalein on neurotransmitters. **(A)** DA, **(B)** DOPAC, **(C)** 5-HT, **(D)** 5-HIAA, **(E)** E, **(F)** GABA, and **(G)** GLU.

Compared with control group, meta-analysis of 12 studies (Im et al., 2005, 2006; Cheng et al., 2008; Li, 2011; Mu et al., 2011; Yu et al., 2012; He et al., 2015; Hu et al., 2016; Hung et al., 2016; Kuang et al., 2017; Yang et al., 2018; Zhao et al., 2018) showed baicalein has significant effects on increasing DA [*n* = 75, SMD = 3.31, 95% CI (2.71–3.90), *P* < 0.00001; heterogeneity: χ = 22.59, df = 11 (*P* = 0.02); *I*^2^ = 51%]. One study (Li, 2011) was removed through sensitivity analysis because baicalin was not administered until 5 weeks after modeling. The remain 11 studies (Im et al., 2005, 2006; Cheng et al., 2008; Mu et al., 2011; Yu et al., 2012; He et al., 2015; Hu et al., 2016; Hung et al., 2016; Kuang et al., 2017; Yang et al., 2018; Zhao et al., 2018) showed significant effects on increasing DA in analysis [*n* = 69, SMD = 3.66, 95% CI (3.01–4.32), *P* < 0.00001; heterogeneity: χ = 16.30, df = 10 (*P* = 0.09); *I*^2^ = 39%; Figure 5A]; Eight studies (Cheng et al., 2008; Li, 2011; Mu et al., 2011; He et al., 2015) for increasing DOPAC [*n* = 49, SMD = 1.89, 95% CI (1.34–2.44), *P* < 0.00001; heterogeneity: χ = 16.26, df = 7 (*P* = 0.02); *I*^2^ = 57%]. Through sensitivity analysis, we removed one study (Yang et al., 2018) because the PD model was established with oxyphenylalanine which was less commonly available. The remain seven studies (Im et al., 2005, 2006; Cheng et al., 2008; Li, 2011; Mu et al., 2011; He et al., 2015; Hu et al., 2016) showed significant effects on increasing DOPAC in analysis [*n* = 41, SMD = 1.70, 95% CI (1.13–2.26), *P* < 0.00001; heterogeneity: χ = 6.84, df = 6 (*P* = 0.34); *I*^2^ = 12%; Figure 5B]; Meta-analysis of 2 studies (Cheng et al., 2008; Li, 2011) increasing the level of 5-HT [*n* = 12, SMD = 2.12, 95% CI (1.02–3.23), *P* < 0.00001; heterogeneity: χ = 1.19, df = 1 (*P* = 0.28); *I*^2^ = 16%; Figure 5C]; Meta-analysis of 2 studies (Cheng et al., 2008; Li, 2011) increasing the level of 5-HIAA [*n* = 12, SMD = 1.45, 95% CI (0.49–2.42), *P* < 0.00001; heterogeneity: χ = 1.39, df = 1 (*P* = 0.24); *I*^2^ = 28%; Figure 5D]; Meta-analysis of 2 studies (Li, 2011; Yang et al., 2018) showed insignificant effect of baicalein for increasing the level of E in PD animals compared with control group [*n* = 14, SMD = 0.47, 95% CI (0.29–1.23), *P* < 0.00001; heterogeneity: χ = 0.53, df = 1 (*P* = 0.47); *I*^2^ = 0%; Figure 5E]; Meta-analysis of 2 studies (Li, 2011; Yu et al., 2012) increasing the level of GABA [*n* = 12, SMD = 5.57, 95% CI (3.44–7.70), *P* < 0.00001; heterogeneity: χ = 1.42, df = 1 (*P* = 0.23); *I*^2^ = 29%; Figure 5F]; Meta-analysis of 2 studies (Li, 2011; Yu et al., 2012) increasing the level of GLU [*n* = 12, SMD = −2.47, 95% CI (−3.67 to −1.27), *P* < 0.00001; heterogeneity: χ = 1.68, df = 1 (*P* = 0.19); *I*^2^ = 41%; Figure 5G].

##### The inhibition of oxidative stress

**Figure 6:**
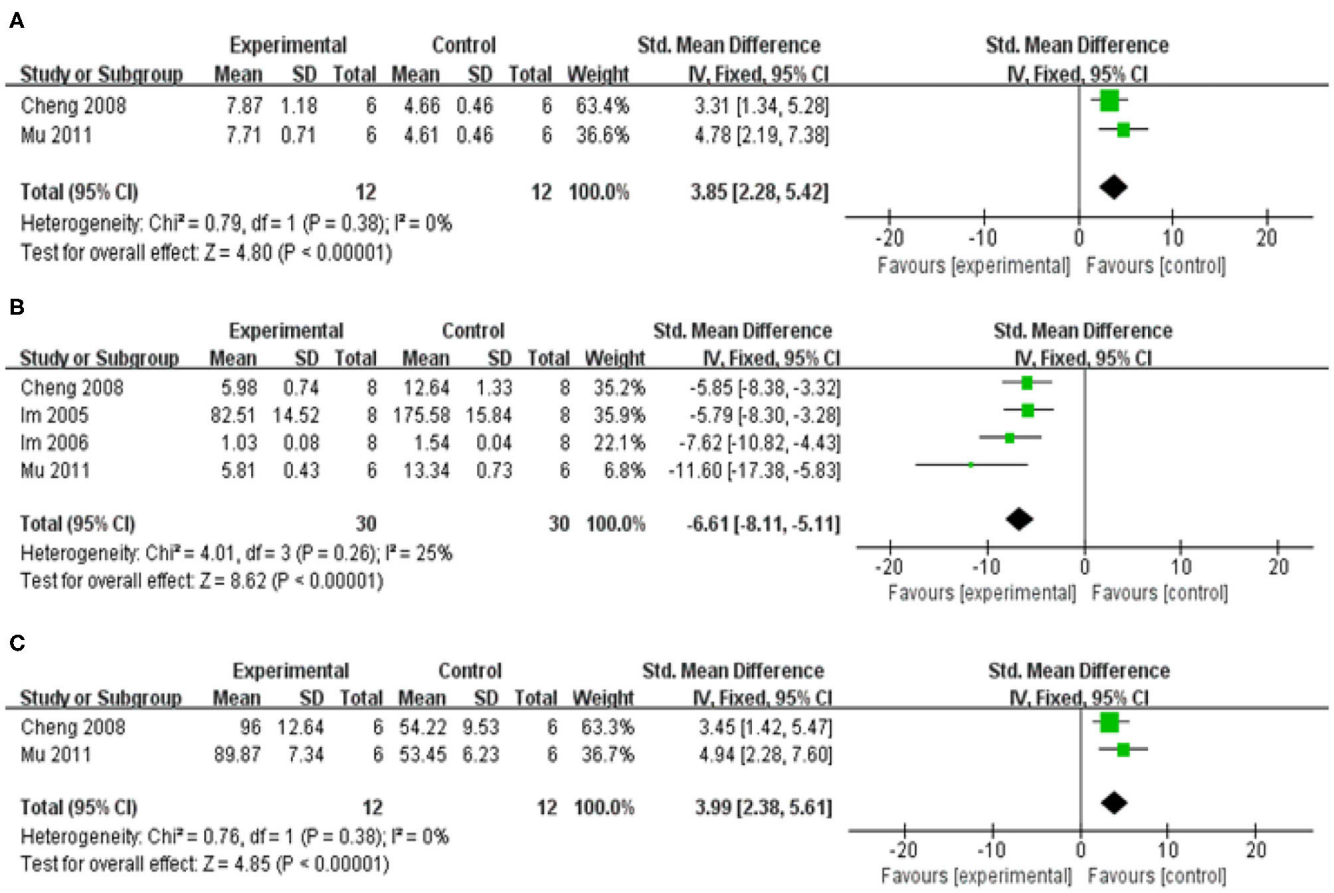
Forest plot of studies investigating the effect of baicalein on the oxidative stress. **(A)** SOD, **(B)** MDA, and **(C)** GSH-Px.

Compared with control group, meta-analysis of two studies (Cheng et al., 2008; Mu et al., 2011) showed baicalein has significant effects on increasing SOD [*n* = 12, SMD = 3.85, 95% CI (2.28–5.42), *P* < 0.00001; heterogeneity: χ = 0.79, df = 1 (*P* = 0.38); *I*^2^ = 0%; Figure 6A]; Meta-analysis of five studies (Im et al., 2005, 2006; Cheng et al., 2008; Mu et al., 2011; Yang et al., 2018) showed baicalein has significant effects on decreasing MDA [*n* = 38, SMD = −3.62, 95% CI (−4.55 to −2.68), *P* < 0.00001; heterogeneity: χ = 28.91, df = 4 (*P* < 0.00001); *I*^2^ = 86%]; Through sensitivity analysis, we removed one study (Yang et al., 2018) because of the same reason mentioned above. The remain four studies (Im et al., 2005, 2006; Cheng et al., 2008; Mu et al., 2011) showed significant effects on decreasing MDA in analysis [*n* = 30, SMD = −6.61, 95% CI (−8.11 to −5.11), *P* < 0.00001; heterogeneity: χ = 4.01, df = 3 (*P* = 0.26); *I*^2^ = 25%; Figure 6B]; Meta-analysis of three studies (Cheng et al., 2008; Mu et al., 2011; Yang et al., 2018) showed baicalein has significant effects on increasing GSH-Px [*n* = 20, SMD = 0.54, 95% CI (−0.34 to 1.42); heterogeneity: χ = 25.75, df = 2 (*P* < 0.00001); *I*^2^ = 92%]; We used sensitivity analyses omitting one study at a time. And one study (Yang et al., 2018) was removed because of the same reason mentioned above. The remain two studies (Cheng et al., 2008; Mu et al., 2011) showed significant effects on increasing GSH-Px in analysis [*n* = 12, SMD = 3.99, 95% CI (2.38–5.61), *P* < 0.00001; heterogeneity: χ = 0.76, df = 1 (*P* =0.38); *I*^2^ = 0%; Figure 6C].

##### Regulation of enzyme activity

**Figure 7:**
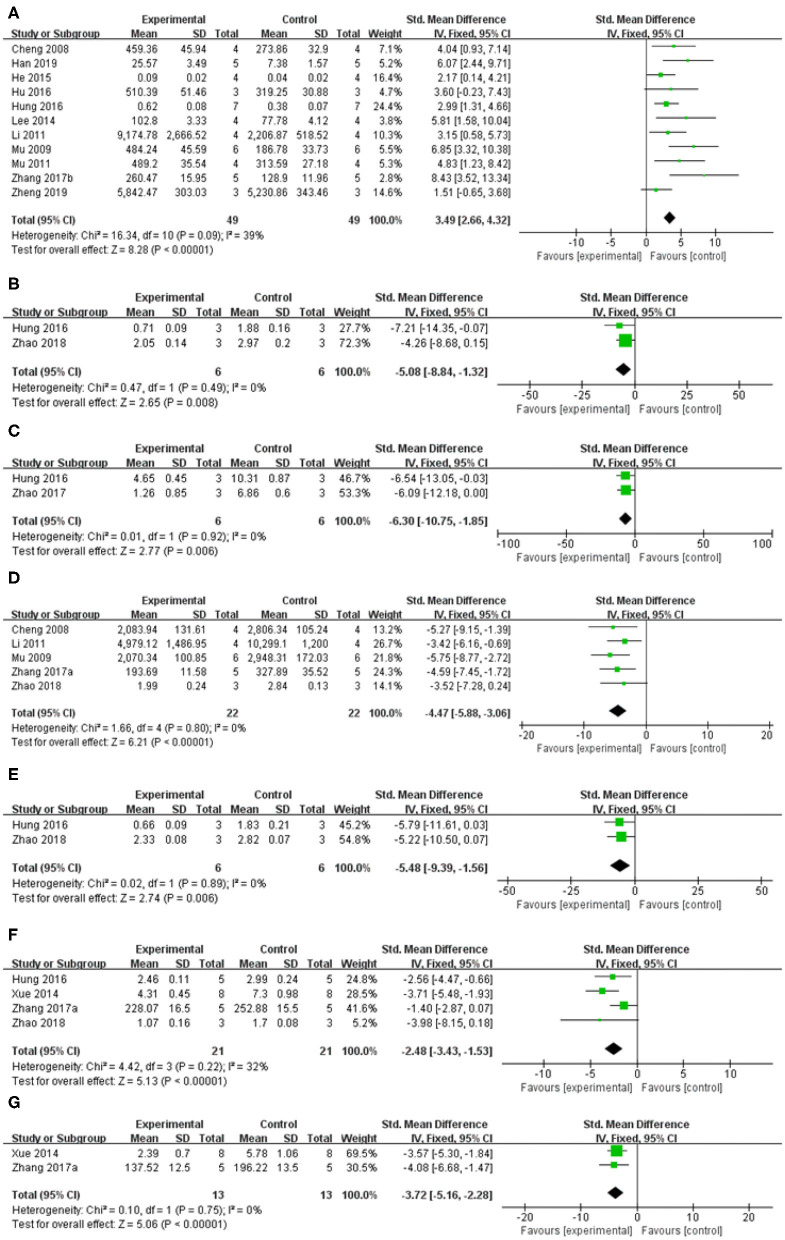
Forest plot: effects of baicalein for the change of **(A)** TH, **(B)** Cathepsin B, **(C)** ED-1, **(D)** GFAP, **(E)** a-Caspase-1, **(F)** IL-1β, and **(G)** TNF-α compared with control group.

Meta-analysis of 13 studies (Im et al., 2005, 2006; Cheng et al., 2008; Mu et al., 2009; Mu et al., 2011; Li, 2011; Lee et al., 2014; He et al., 2015; Hu et al., 2016; Hung et al., 2016; Zhang et al., 2017b; Han et al., 2019; Zheng et al., 2019) showed baicalein had significant effect on increasing the level of TH compared with the control group [*n* = 63, SMD: 3.80, 95% CI (2.98–4.62), *P* < 0.00001; heterogeneity: χ = 41.40, df = 12 (*P* < 0.00001); *I*^2^ = 71%]. Owing to the obvious heterogeneity, we conducted a sensitivity analyses and removed two study (Im et al., 2005, 2006) that Parkinson's model of ICR mice was induced with 6-OHDA. Meta-analysis of the remaining 11 studies (Cheng et al., 2008; Mu et al., 2009; Mu et al., 2011; Li, 2011; Lee et al., 2014; He et al., 2015; Hu et al., 2016; Hung et al., 2016; Zhang et al., 2017b; Han et al., 2019; Zheng et al., 2019) showed baicalein had significant effect on increasing the level of TH compared with the control group [*n* = 49, SMD: 3.49, 95% CI (2.66–4.32), *P* < 0.00001; heterogeneity: χ = 16.34, df = 10 (*P* = 0.09), *I*^2^ = 39%; Figure 7A]. One study (Yu et al., 2012) demonstrated that baicalein increased the GS activity (*P* < 0.05), while reduced the GABA-T activity (*P* < 0.05) compared with the control group. One study (Yang et al., 2018) increased the AchE activity (*P* < 0.05).

##### The inhibition of neuroinflammation

Meta-analysis of 4 studies (Xue et al., 2014; Hung et al., 2016; Zhang et al., 2017a; Zhao et al., 2018) indicated baicalein was significant for decreasing the level of Interleukin-1β (IL-1β) compared with control group [*n* = 21, SMD = −2.48, 95% CI (−3.43 to −1.53), *P* < 0.00001; heterogeneity: χ = 4.42, df = 3 (*P* = 0.22); *I*^2^ = 32%; Figure 7F]. Meta-analysis of 2 studies (Xue et al., 2014; Zhang et al., 2017a) decreasing expression of tumor necrosis factor (TNF-a) [*n* = 13, SMD = −3.72, 95% CI (−5.16 to −2.28), *P* < 0.00001; heterogeneity: χ = 0.10, df = 1 (*p* = 0.75); *I*^2^ = 0%; Figure 7G]. Meta-analysis of 2 studies (Hung et al., 2016; Zhao et al., 2018) showed baicalein was significant for decreasing the level of Cathepsin B [*n* = 6, SMD = −5.08, 95% CI (−8.84 to −1.32), *P* = 0.008; heterogeneity: χ = 0.47, df = 1 (*P* = 0.49); *I*^2^ = 0%; Figure 7B], and ED-1 [*n* = 6, SMD = −6.30, 95% CI [−10.75 to −1.85], *P* = 0.006; heterogeneity: χ = 0.01, df = 1 (*P* = 0.92); *I*^2^ = 0%; Figure 7C] compared with control group. Meta-analysis of 5 studies (Cheng et al., 2008; Mu et al., 2009; Li, 2011; Zhang et al., 2017a; Zhao et al., 2018) showed baicalein was significant for decreasing the level of GFAP compared with control group [*n* = 22, SMD = −4.47, 95% CI (−5.88 to −3.06), *P* < 0.00001; heterogeneity: χ = 1.66, df = 4 (*p* = 0.80); *I*^2^ = 0%; Figure 7D]. Two studies (Zhang et al., 2017a; Zheng et al., 2019) for decreasing expression of IL-6. 1 study (Zhang et al., 2017a) for decreasing expression of p-IkB/IkB, p-p65/p65, p-p38/p38, and p-Erk1/2/Erk1/2 (*P* < 0.05), while increasing expression of p-JNK/JNK (*P* < 0.05).

##### The inhibition of neuronal apoptosis

Meta-analysis of 2 studies (Hung et al., 2016; Zhao et al., 2018) indicated baicalein was significant for decreasing the expression of a-Caspase 1 compared with control group [*n* = 6, SMD = −5.48, 95% CI (−9.39 to −1.56), *P* = 0.006; heterogeneity: χ = 0.02, df = 1 (*P* = 0.89); *I*^2^ = 0%; Figure 7E]. One study (Hung et al., 2016) for decreasing protein expression of a-Caspase 9 (*P* < 0.05) and a-Caspase 12 (*P* < 0.05). One study (Zhao et al., 2018) for decreasing protein expression of a-Caspase 3 (*P* < 0.05), RIPK-1 (*P* < 0.05), and RIPK-3 (*P* < 0.05). One study (Zheng et al., 2019) for decreasing expression of Bax mRNA (*P* < 0.05).

##### The restoration of mitochondrial dysfunction

One study (Zhang et al., 2017b) for increasing the protein levels of PGC-1α (*P* < 0.05), NRF-1 (*P* < 0.05), TRAM (*P* < 0.05), and the activity of mitochondrial complex I (*P* < 0.05) and ATP levels (*P* < 0.01) in the ventral midbrain in rotenone-induced PD rats.

##### The inhibition of abnormal protein aggregation

One study (Hu et al., 2016) showed that baicalein decreased α-Synuclein (α-syn)levels in the ileum and thoracic spinal cord in the rotenone induced PD mouse model.

##### The prevention of MPP^+^-induced autophagy

One study (Hung et al., 2016) showed that baicalein inhibited MPP^+^-induced elevation in light chain 3-II (LC3-II) level in the rat nigrostriatal dopaminergic system.

### Publication Bias

**Figure 8:**
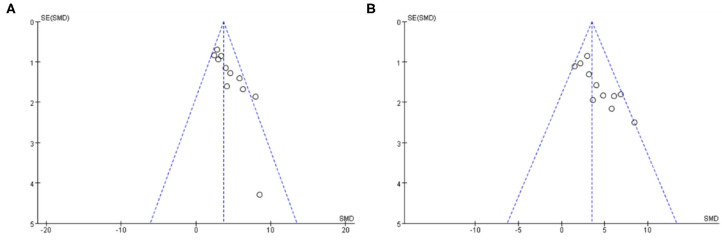
The funnel plot: effects of baicalein for increasing DA **(A)** and TH **(B)**.

Funnel plots were reviewed for two outcomes about DA and TH (Figure 8). The funnel graph revealed an asymmetry distribution of included studies. The Egger's regression (*p* < 0.05) also confirmed the publication bias due to a small number of studies reporting negative Baicalein treatment effects. Although the Trim and Fill methods were used to correct publication bias, the results did not change.

## Discussion

### Summary of Results

To our knowledge, this is the first preclinical evidences to determine the effects of baicalein for experimental PD in mice and rats. The findings available from the present study showed that baicalein could improve behavior function in experimental PD, mainly through the mechanisms of adjusting neurotransmitters, regulating enzyme activities, suppressing oxidative stress, ameliorating mitochondrial dysfunction, restraining neuroinflammation, inhibiting abnormal protein aggregation, and neuronal apoptosis.

### Limitations

This meta-analysis had several limitations. First, all the databases we searched were in English or Chinese, leading to certain deviations. Second, negative findings are less likely to be published, which may overestimate the true efficacy of baicalein to a certain degree. Third, the methodological quality of included studies was considered moderate, which was an inherent drawback in the primary study. In particular, all the studies failed to mention the allocation concealment, blinding of outcome assessment, etc. Fourth, the high heterogeneity among studies was possibly associated with different conditions including different models of PD induction, different administration route, and different doses of neurotoxins and baicalein. Thus, the results in this study should be partially treated with caution.

### Implications for Practice

Currently, the medical animal experiments have become an important means of biomedical research which link basic research and clinical trials. Due to the limitations of medical ethics, some trauma researches, toxicological characteristics, and pharmacodynamics studies are not suitable for human clinical trials. Therefore, animal models capable of replicating important functional, structural, and molecular pathological features of human disease to the maximum extent are essential for clinical translation (Saulnier-Blache et al., 2018).

**Figure 9:**
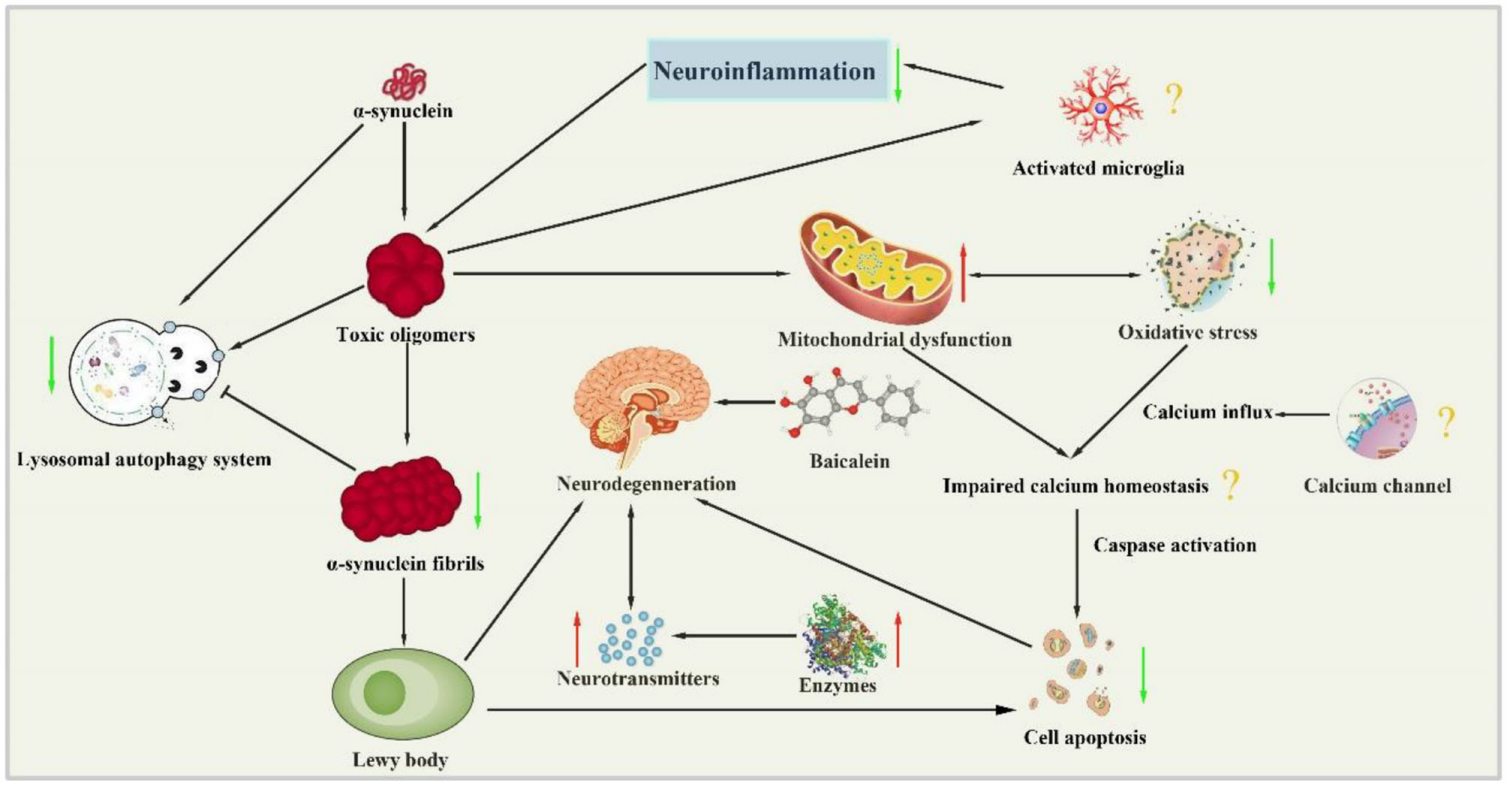
Neuroprotection mechanisms of baicalein in PD animal model. (↑, upregulation; ↓, downregulation; ?, unknown mechanism).

The present study demonstrated that baicalein had neuroprotective effects in PD models according to the neurobehavioral. The mechanisms of baicalein for PD are summarized as follows: (1) Correction of neurotransmitters: the pathological mechanisms of PD is closely related to the abnormal release of various neurotransmitters in brain, including monoamine neurotransmitters such as DA, DOPAC, NE and 5-HT, amino acid neurotransmitters such as Glu, γ-GABA and ACh, and peptide transmitters etc (Kulikova et al., 2018). In central nervous system, the main form of synaptic transmission is neurochemical transmission. The neurotransmitters released by presynaptic membrane can bind with the corresponding postsynaptic membrane receptors, and result in producing synaptic depolarization potential or hyperpolarization potential, which weaken or enhance excitability of postsynaptic neurons (Ztaoua and Amalric, 2019). Interestingly, the two chemical transmitters, DA and Ach are antagonistic to each other in the corresponding nerve cells. Although the level of Ach is normal in the brain of patients with PD, the decrease of DA content leads to the relative hyperactivity of cholinergic neurons, which causes some symptoms of PD (McKinley et al., 2019). The evidence available from the present study showed that baicalein could effectively rectify the content of monoamine transmitters and amino acid transmitters in animals with PD. (2) Regulation of enzyme activity: as we all know, the secretion of neurotransmitters in brain must be affected by the activity of corresponding enzyme. TH, as the rate-limiting enzyme in the synthesis process of DA, is a specific marker of dopaminergic neurons (Liang et al., 2015). AchE is an essential enzyme for the selective hydrolysis of ACh, which can hydrolyze ACh into choline and acetic acid (Karumuri et al., 2019). GABA and GLu are metabolized by continuous action of GABA transaminase (GABA-T) and glutamine synthetase (GS) protein (Baber and Haghighat, 2010). Our data indicated that baicalein could balance neurotransmitter by regulating the activity of TH, AchE, GABA-T, and GS. (3) Anti-oxidative stress: oxidative stress plays a key role in the occurrence and development of PD. The content of MDA is an important indicator of the level of oxygen free radicals, which can indirectly reflect the degree of cell damage. As the main free radical scavenging enzyme in the body, SOD reflects the endogenous antioxidant capacity of the organism. And GSH-Px is a critical enzyme widely used in the body that can catalyze the decomposition of H_2_O_2_ (Sharma et al., 2020). Our data demonstrated that baicalein had anti-oxidative stress effects by increasing the activity of antioxidant enzymes in brain tissues, improving the antioxidant capacity, scavenging free radicals, inhibiting lipid peroxidation, and protecting the structural and functional integrity of cell membranes. (4) Anti-inflammation: neuroinflammation is an intricate cascade of neurodegenerative changes in Parkinson's syndrome, including the activation of microglia and astrocytes and the release of inflammatory cytokines. NF-κB is widely expressed in microglia, astrocytes and neurons. In normal state, the endogenous inhibitor IκB inactivate NF-κB, while IL-1β and TNF-α can induce the phosphorylation and degradation of IKK-β, which translocate NF-κB into the nucleus and promote the expression of inflammatory genes (Yan et al., 2019; Wang et al., 2020). The pooled data showed that treatment of baicalein prominently suppressed the expression of NF-κB, GFAP (a biomarker of activated astrocytes), ED-1 (a biomarker of activated microglia), and mature cathepsin B (a cysteine lysosomal protease) (Zhao et al., 2018). (5) Restoration of mitochondrial dysfunction: mitochondrial dysfunction is an early signal in almost all neurodegenerative diseases, including PD (Angelova et al., 2018). Baicalein greatly increased the expression of the key regulators of mitochondrial biogenesis (PGC-1α, NRF-1, and TFAM). In addition, baicalein could maintain the function of mitochondria by partially enhancing the activity of mitochondrial complex enzyme in brain, and ultimately delay the progression of PD. (6) The inhibition of abnormal protein aggregation: one of the characteristics of pathological changes in PD is the appearance of Lewy bodies which are composed of α-syn (Reuland and Church, 2020). Furthermore, the mutation or overexpression of α-syn gene can accelerate mitochondrial dysfunction, enhance sensitivity to oxidative stress and increase DAT-mediated toxicity, thus promoting cell death (Vekrellis et al., 2011). Our study indicated that baicalein could inhibit the formation of α-syn oligomers and subsequently prevent the progression of α-syn accumulation *in vivo*. (7) Antiapoptosis: Growing evidence suggests that another possibility for dopaminergic neuron loss is the abnormal occurrence of apoptosis (Liu et al., 2019). The caspases are a class of cysteine proteases, many of whose members are involved in apoptosis. Caspases convey the apoptotic signal through proteolytic cascade with caspases cleaving and subsequently activate other caspases that degrade cellular targets leading to cell death. Additionally, Bcl-2 family which is a core member of apoptosis gene families plays a critical role in apoptotic process (Schulz and Gerhardt, 2001). (8) Regulation of autophagy: Autophagy is a lysosome-mediated degradation process that involves degradation of redundant or defective cellular components within the cell, including both misfolded proteins and damaged organelles (Hou et al., 2020). Thus, autophagy activity is correlated with disease progression in neurodegenerative disorders such as AD and PD (Nobuhiro et al., 2018). However, dysregulated or excessive autophagy could cause autophagic cell death, the type II programmed cell death (Thellung et al., 2019). LC3-II is currently considered to be a biomarker of autophagy, which reflect the extent of autophagy (Runwal et al., 2019). Systemic administration of baicalein for 2 days significantly attenuated MPP^+^-induced elevation in LC3-II in the infused substantia nigra (Hung et al., 2016). Our study indicates that baicalein prevents programmed cell death, mainly by regulating the expression of genes related to apoptosis and autophagy. To summarize, we present a schematic overview of the neuroprotective mechanisms of baicalein in PD (Figure 9).

## Conclusion

The present study showed baicalein could exert potential neuroprotective effects in experimental PD, largely through mechanisms involving antioxidation, anti-inflammatory, regulating neurotransmitters, adjusting enzyme activity, inhibiting protein aggregation, restorating mitochondrial dysfunction, inhibiting apoptosis, and autophagy. Thus, baicalein could be a candidate for further clinical trials of PD.

## Author Contributions

YW and XL conceived this review and completed the manuscript. YW, NW, and XL performed the literature searches electronically and manually. All authors contributed to the article and approved the submitted version.

## Conflict of Interest

The authors declare that the research was conducted in the absence of any commercial or financial relationships that could be construed as a potential conflict of interest.

## References

[B1] Angelova P. R. Barilani M. Lovejoy C. Dossena M. Viganò M. Seresini A.. (2018). Mitochondrial dysfunction in Parkinsonian mesenchymal stem cells impairs differentiation. Redox. Biol. 14, 474–484. 10.1016/j.redox.2017.10.016 29096320 PMC5680522

[B2] Baber Z. Haghighat N. (2010). GLU synthetase gene expression and GLU transporters in C6-glioma cells. Metab. Brain Dis. 25, 413–418. 10.1007/s11011-010-9223-9 21107897

[B3] Cheng Y. X. He G. R. Mu X. Zhang T. T. Li X. X. Hu J. J.. (2008). Neuroprotective effect of baicalein against MPTP neurotoxicity: behavioral, biochemical and immunohistochemical profile. Neurosci. Lett. 441, 16–20. 10.1016/j.neulet.2008.05.116 18586394

[B4] Gao L. Li C. Yang R. Y. Lian W. W. Fang J. S. Pang X. C.. (2015). Ameliorative effects of baicalein in MPTP-induced mouse model of parkinson's disease: a microarray study. Pharmacol. Biochem. Behav. 133, 155–163. 10.1016/j.pbb.2015.04.004 25895692

[B5] Han F. Wang J. Li Y. L. (2019). Protection of baicalein combined with rifampicin on substantia nigra neurons of rotenone-induced Parkinson's disease in rats. *J*. Toxicol. 33, 27–33. 10.16421/j.cnki.1002-3127.2019.01.005

[B6] He G. R. Mu X. Li X. X. Wang Y. H. Fang L. H. Sun L. (2015). Effect of baicalein on brain injury induced by 6-hydroxydopamine at different sites in rats. Chin. Pharmacol. Bull. 31, 623–630. 10.3969/j.issn.1001-1978.2015.05.008

[B7] Hooijmans C. R. Rovers M. M. de Vries R. B. Leenaars M. Ritskes-Hoitinga M. Langendam M. W. (2014). SYRCLE's risk of bias tool for animal studies. BMC. Med. Res. Methodol. 14:43. 10.1186/1471-2288-14-43 24667063 PMC4230647

[B8] Hou X. Watzlawik J. O. Fiesel F. C. Springer W. (2020). Autophagy in Parkinson's Disease. J. Mol. Biol. 432, 2651–2672. 10.1016/j.jmb.2020.01.037 32061929 PMC7211126

[B9] Hu Q. Uversky V. N. Huang M. Y. Kang H. C. Xu F. Liu X. Y.. (2016). Baicalein inhibits α-synuclein oligomer formation and prevents progression of α-synuclein accumulation in a rotenone mouse model of Parkinson's disease. Biochim. Biophys. Acta. 1862, 1883–1890. 10.1016/j.bbadis.2016.07.008 27425033

[B10] Hung K. C. Huang H. J. Wang Y. T. Lin A. M. (2016). Baicalein attenuates α-synuclein aggregation, inflammasome activation and autophagy in the MPP^+^-treated nigrostriatal dopaminergic system *in vivo*. J. Ethnopharmacol. 194, 522–529. 10.1016/j.jep.2016.10.040 27742410

[B11] Im H. I. Joo W. S. Nam E. Lee E. S. Hwang Y. J. Kim Y. S. (2005). Baicalein prevents 6-hydroxydopamine-induced dopaminergic dysfunction and lipid peroxidation in mice. J. Pharmacol. Sci. 98, 185–189. 10.1254/jphs.SC0050014 15942123

[B12] Im H. I. Nam E. Lee E. S. Hwang Y. J. Kim Y. S. (2006). Baicalein protects 6-OHDA-induced neuronal damage by suppressing oxidative stress. Korean. J. Physiol. Pharmacol. 10, 309–315.

[B13] Karumuri S. B. Singh H. Naqvi S. Mishra A. Flora S. J. S. (2019). Impact of chronic low dose exposure of monocrotophos in rat brain: Oxidative/nitrosative stress, neuronal changes and cholinesterase activity. Toxicol. Rep. 6, 1295–1303. 10.1016/j.toxrep.2019.11.005 31867220 PMC6906705

[B14] Kovács M. Makkos A. Pintér D. Juhász A. Darnai G. Karádi K.. (2019). Screening for problematic internet use may help identify impulse control disorders in Parkinson's disease, Behav. Neurol. 2019:4925015. 10.1155/2019/4925015 30863462 PMC6378069

[B15] Kuang L. H. Cao X. B. Lu Z. N. (2017). Baicalein protects against rotenone-induced neurotoxicity through induction of autophagy. Biol. Pharm. Bull. 40, 1537–1543. 10.1248/bpb.b17-00392 28659545

[B16] Kulikova O. I. Berezhnoy D. S. Stvolinsky S. L. Lopachev A. V. Orlova V. S. Fedorova T. N.. (2018). Neuroprotective effect of the carnosine- α-lipoic acid nanomicellar complex in a model of early-stage Parkinson's disease. Regul. Toxicol. Pharmacol. 95, 254–259. 10.1016/j.yrtph.2018.03.025 29601911

[B17] Lee E. Park H. R. Ji S. T. Lee Y. Lee J. (2014). Baicalein attenuates astroglial activation in the 1-methyl-4-phenyl-1,2,3,4-tetrahydropyridine-induced Parkinson's disease model by downregulating the activations of nuclear factor-κB, ERK, and JNK. J. Neurosci. Res. 92, 130–139. 10.1002/jnr.23307 24166733

[B18] LeWitt P. A. Fahn S. (2016). Levodopa therapy for Parkinson disease: a look backward and forward. Neurology 86(14 Suppl. 1), S3–S12. 10.1212/WNL.0000000000002509 27044648

[B19] Li X. X. (2011). Experimental Study of the Therapeutic Effect and Mechanism of Baicalein on Parkinson's Disease. Bei Jing: Chinese Academy of Medical Sciences & Peking Union Medical College.

[B20] Liang Y. R. Jing X. N. Zeng Z. F. Bi W. Chen Y. Wu X.. (2015). Rifampicin attenuates rotenone-induced inflammation via suppressing NLRP3 inflammasome activation in microglia. Brain Res. 1622, 43–50. 10.1016/j.brainres.2015.06.008 26086368

[B21] Liu J. Q. Chu S. F. Zhou X. Zhang D. Y. Chen N. H. (2019). Role of chemokines in Parkinson's disease. Brain Res. Bull. 152, 11–18. 10.1016/j.brainresbull.2019.05.020 31136787

[B22] Martin I. Dawson V. L. Dawson T. M. (2011). Recent advances in the genetics of Parkinson's disease. Annu. Rev. Genomics Hum. Genet. 12, 301–325. 10.1146/annurev-genom-082410-101440 21639795 PMC4120236

[B23] McKinley J. W. Shi Z. Q. Kawikova I. Hur M. Bamford I. J. Devi S. P. S.. (2019). Dopamine deficiency reduces striatal cholinergic interneuron function in models of Parkinson's disease. Neuron 103, 1056–1072. 10.1016/j.neuron.2019.06.013 31324539 PMC7102938

[B24] Moher D. Shamseer L. Clarke M. Ghersi D. Liberati A. Petticrew M.. (2015). Preferred reporting items for systematic review and meta-analysis protocols (PRISMA-P) 2015 statement. *Syst*. Rev. 4:1. 10.1186/2046-4053-4-1 25554246 PMC4320440

[B25] Mu X. He G.R. Yuan X. Li X.X. Du G.H. (2011). Baicalein protects the brain against neuron impairments induced by MPTP in C57BL/6 mice. Pharmacol. Biochem. Behav. 98, 286–291. 10.1016/j.pbb.2011.01.011 21262257

[B26] Mu X. He G. R. Cheng Y. X. Li X. X. Xu B. Du G. H. (2009). Baicalein exerts neuroprotective effects in 6-hydroxydopamine-induced experimental parkinsonism *in vivo* and *in vitro*. Pharmacol. Biochem. Behav. 92, 642–648. 10.1016/j.pbb.2009.03.008 19327378

[B27] Naskar A. Manivasagam T. Chakraborty J. Singh R. Thomas B. Dhanasekaran M.. (2013). Melatonin synergizes with low doses of L-DOPA to improve dendritic spine density in the mouse striatum in experimental parkinsonism. J. Pineal. Res. 55, 304–312. 10.1111/jpi.12076 23952687

[B28] Nobuhiro F. Minkyoung S. Shigeomi S. (2018). Association between autophagy and neurodegenerative diseases. Front. Neurosci. 12:255 10.3389/fnins.2018.00255 29872373 PMC5972210

[B29] Reuland C. J. Church F. C. (2020). Synergy between plasminogen activator inhibitor-1, α-synuclein, and neuroinflammation in Parkinson's disease. Med. Hypotheses. 138:109602. 10.1016/j.mehy.2020.109602 32035284

[B30] Runwal G. Stamatakou E. Siddiqi F. H. Puri C. Zhu Y. Rubinsztein D. C. (2019). LC3-positive structures are prominent in autophagy-deficient cells. Sci Rep. 9:10147. 10.1038/s41598-019-46657-z 31300716 PMC6625982

[B31] Saulnier-Blache J. S. Rory W. Kristaps K. Delyth G. Ioana A. Kastenmüller G.. (2018). Ldlr-/- and ApoE-/- mice better mimic the human metabolite signature of increased carotid intima media thickness compared to other animal models of cardiovascular disease. Atherosclerosis. 276, 140–147. 10.1016/j.atherosclerosis.2018.07.024 30059845

[B32] Schapira A. H. V. Chaudhuri K. R. Jenner P. (2017). Non-motor features of Parkinson disease. Nat. Rev. Neurosci. 18:509 10.1038/nrn.2017.91 28720825

[B33] Schindlbeck K. A. Eidelberg D. (2018). Network imaging biomarkers: insights and clinical applications in parkinson's disease. Lancet. Neurol. 17, 629–640. 10.1016/S1474-4422(18)30169-8 29914708

[B34] Schulz J. B. Gerhardt E. (2001). Apoptosis: its relevance to Parkinson's disease. Clin. Neurosci. Res. 1, 427–433. 10.1016/S1566-2772(01)00021-4

[B35] Sena E. S. Currie G. L. McCann S. K. Macleod M. R. Howells D. W. (2014). Systematic reviews and meta-analysis of preclinical studies: why perform them and how to appraise them critically. J. Cereb. Blood Flow Metab. 34, 737–742. 10.1038/jcbfm.2014.28 24549183 PMC4013765

[B36] Sharma S. Rabbani S. A. Narang J. K. Pottoo F. H. Ali J. Kumar S.. (2020). Role of rutin nanoemulsion in ameliorating oxidative stress: pharmacokinetic and pharmacodynamics studies. Chem. Phys. Lipids. 228:104890. 10.1016/j.chemphyslip.2020.104890 32032570

[B37] Sowndhararajan K. Deepa P. Kim M. Park S. J. Kim S. (2017). Baicalein as a potent neuroprotective agent: a review, Biomed Pharmacother. 95, 1021–1032. 10.1016/j.biopha.2017.08.135 28922719

[B38] Thellung S. Corsaro A. Nizzari M. Barbieri F. Florio T. (2019). Autophagy activator drugs: a new opportunity in neuroprotection from misfolded protein toxicity. Int. J. Mol. Sci. 20:901. 10.3390/ijms20040901 30791416 PMC6412775

[B39] Vekrellis K. Xilouri M. Emmanouilidou E. Rideout H. J. Stefanis L. (2011). Pathological roles of α-synuclein in neurological disorders. Lancet Neurol. 10, 1015–1025. 10.1016/S1474-4422(11)70213-7 22014436

[B40] Wang H. Naghavi M. Allen C. Barber R. M. Bhutta Z. A. Carter A.. (2016). Global, regional, and national life expectancy, all-cause mortality, and cause specific mortality for 249 causes of death, 1980-2015: a systematic analysis for the global burden of disease study. Lancet 388, 1459–1544. 10.1016/S0140-6736(16)31012-1 27733281 PMC5388903

[B41] Wang Z. S. Dong H. T. Wang J. H. Huang Y. L. Zhang X. S. Tang Y. L.. (2020). Pro-survival and anti-inflammatory roles of NF-κB c-Rel in the Parkinson's disease models. Redox. Biol. 30:101427. 10.1016/j.redox.2020.101427 31986466 PMC6994410

[B42] Xue X. H. Liu H. Qi L. F. Li X. L. Guo C. J. Gong D. R.. (2014). Baicalein ameliorated the upregulation of striatal glutamatergic transmission in the mice model of Parkinson's disease. Brain Res. Bull. 103, 54–59. 10.1016/j.brainresbull.2014.02.004 24576689

[B43] Yan T. X. Sun Y. Y. Gong G. W. Li Y. Fan K. Y. Wu B.. (2019). The neuroprotective effect of schisandrol A on 6-OHDA-induced PD mice may be related to PI3K/AKT and IKK/IκBα/NF-κB pathway. Exp. Gerontol. 128:110743. 10.1016/j.exger.2019.110743 31629801

[B44] Yang Y. L. Zhang X. Zhang W. Wang H. G. Zhao X. Y. Song J. K. (2018). Inhibitory effect of baicalein on mice tremor induced by oxotremorine and mechanisms. Chinese J. New Drugs 27, 914–920.

[B45] Yu X. He G. R. Sun L. Lan X. Shi L. L. Xuan Z. H.. (2012). Assessment of the treatment effect of baicalein on a model of parkinsonian tremor and elucidation of the mechanism. Life Sci. 91, 5–13. 10.1016/j.lfs.2012.05.005 22634324

[B46] Zhang X. Du L. D. Zhang W. Yang Y. L. Zhou Q. M. Du G. H. (2017a). Therapeutic effects of baicalein on rotenone-induced Parkinson's disease through protecting mitochondrial function and biogenesis. Sci Rep. 7:9968. 10.1038/s41598-017-07442-y 28855526 PMC5577282

[B47] Zhang X. Yang Y. L. Du L. D. Zhang W. Du G. H. (2017b). Baicalein exerts anti-neuroinflammatory effects to protect against rotenone induced brain injury in rats. Int. Immunopharmacol. 50, 38–47. 10.1016/j.intimp.2017.06.007 28623717

[B48] Zhao W. Z. Wang H. T. Huang H. J. Lo Y. L. Lin M. Y. (2018). Neuroprotective effects of baicalein on acrolein-induced neurotoxicity in the nigrostriatal dopaminergic system of rat brain. Mol. Neurobiol. 55, 130–137. 10.1007/s12035-017-0725-x 28866823

[B49] Zheng Z. V. Cheung C. Y. Lyu H. Chan H. Y. Li Y. Bian Z. X.. (2019). Baicalein enhances the effect of low dose Levodopa on the gait deficits and protects dopaminergic neurons in experimental Parkinsonism. J. Clin. Neurosci. 64, 242–251. 10.1016/j.jocn.2019.02.005 30905662

[B50] Ztaoua S. Amalric M. (2019). Contribution of cholinergic interneurons to striatal pathophysiology in Parkinson's disease. Neurochem. Int. 126, 1–10. 10.1016/j.neuint.2019.02.019 30825602

